# Statistics of the Results of the Capital Operations in the Hospitals of Paris

**Published:** 1842-07-30

**Authors:** M. Clymer


### BIBLIOGRAPHICAL NOTICE.

*Etudes Statistiques sur les résultats des Grandes Opérations dans les Hôpitaux de Paris. De la Mortalité après les Amputations.* Par Mr. MALGAIGNE, Chirurgien de l'hospice de Bicètre.

*Statistics of the results of the Capital Operations in the Hospitals of Paris. Of the Mortality after Amputations.* By M. MALGAIGNE, Surgeon to Bicètre.

[Archives Generales de Médecine, for April and May, 1842, pp. 389 and 50.]

Mr. MALGAIGNE is well known to the profession as the author of a successful treatise on Operative Surgery, as well as a statistical writer of great industry and correctness. His attention was first directed to the subject of the mortality after amputations during the Polish campaign of 1831, where he acted as surgeon to the insurgent army. He commenced operations, he tells us, in the firm expectation of saving at least three out of four of his amputations; a hope, he states, founded on the alleged successes in the hospitals of Paris, during the revolution of July. He amputated immediately and united his stumps. When, on his arrival at Warsaw and Plock, his inquiry after his operations received the uniform answer of *death*! his disappointment was of course immense, and his chagrin proportionate. To console his wounded self-love, he set about, on his return, collecting the real results in the hospitals of Paris; and in publishing them he thinks he will render two important services to the young surgeon—1. That he will be less hasty in his prognosis. 2. That when, after necessary amputations, he shall have, as the rewards of all his care and solicitude, constant reverses, he will be saved the frightful nightmare which pursued our author, and the poignant doubts which he experienced.

From January 1, 1836, to January 1, 1841, 852 amputations were performed in the hospitals of Paris: of the hip-joint 1; of the thigh 201; of the knee-joint 3; of the leg 192; of the foot 38; of the metatarsal bones 8; of the toes 85; total 528, for the inferior extremity. Of the shoulder-joint 14; of the arm 91; of the forearm 28; of the wrist-joint 16; of the carpal bones 9; of the fingers 166; total 324, for the superior extremity. The mortality was 332, or nearly two in five; 255 for the inferior extremity, or about one-half; 77 for the superior extremity, or nearly one-fourth.

The single coxo-femoral disarticulation was secondary, for a gunshot wound, and on a male patient of 21 years, and was fatal.* In 201 amputations of the thigh in its continuity, including all causes, ages, and both sexes, there were 126 deaths, nearly 62 per cent., or about two-thirds. For wounds, burns, fractures, &c., there were 46; 44 men, and 2 women; of the men, 34 died and 10 recovered; of the women, both recovered. Total of deaths 34, or three-fourths. For chronic diseases there were 153 amputations, 92 deaths, 61 recoveries, or 3 deaths in 5. The number is completed by two amputations for double anchylosis, performed successively by M. Velpeau, on a man of 28 years, and with success.†

*Amputation of the knee-joint.*—This abandoned operation was revived in 1830 by Mr. Velpeau, who, with his peculiar industry, had collected 14 cases and 13 cures. These were isolated and collected from books, or other imperfect records; and that the conclusions he made were not warrantable,

*The first successful amputation of the hip-joint at Paris was performed in 1841, by Mr. Sedillot, at the military hospital of Val de Grace. In the report made to the French Academy of Medicine on the 3d January on this case, by Baron Larrey, it is recommended to tie the artery before commencing the operation, the utility of this practice having been proved in the course of the practice of the reporter.

† Recently Mr. Jobert, of the Hôpital St. Louis, performed a double amputation of the thigh on a man of 28 years, for a scrofulous tumour of each knee-joint, with entire success.

was conclusively proved by the subsequent constant fatality of the operation, and which, since 1838, has been banished from the hospitals of Paris. Mr. Velpeau has operated 6 times; 4 deaths. Mr. Laugier twice; 2 deaths. Mr. Blandin once; 1 death.

*Amputations of the leg.*—Of these there were 192, and 106 deaths, nearly 55 per cent., or one-ninth less than in amputations of the thigh. For organic diseases 112, 55 deaths, or one-half. For traumatic lesions 79, 50 deaths, or nearly two-thirds. One amputation of *complaisance*, performed at the Hôtel-Dieu, on a man of 19 years, for club-foot, and followed by death.

*Partial amputations of the foot.*—38 patients had their feet amputated, and 9 died, or 24 per cent. For organic lesions 29, and 3 deaths, or about one-tenth. For traumatic lesions 9, and 6 deaths, or just two-thirds.

*Amputation of the shoulder-joint.*—Surgeons have, in general, reported an incredible success in this operation. Mr. Gouraud, quoted by Mr. Velpeau in his Médecine Opératoire, says, "we have performed and seen performed this operation with such happy results, that we hardly believe it more dangerous than amputation in the continuity of the humerus, and even consider it doubtful whether, in gunshot wounds, it is not to be preferred." M. Blancel mentions 60 successful cases in his thesis. Baron Larrey states that he was successful 90 times in 100 cases. Sabatier speaks in terms of enthusiasm of 14 successes in 17 cases obtained by this surgeon; and Percy states that of 70 cases, one-sixteenth only terminated fatally. Let us see how far M. Malgaigne's statistics confirm these statements. There were in the period of five years under consideration 14 scapulo-humeral disarticulations. In one patient there was at the same time amputation of the thigh, and, as a matter of course, he succumbed. Throwing aside this, there remain 13 cases and 10 deaths. 6 times the operation was performed for chronic diseases; of these 4 were men from 15 to 41 years; the young man of 15 recovered, all the others died. Women, respectively of 19 and 40 years of age, 2; both recovered. For traumatic lesions 7 operations, all males, from 27 to 65 years; 7 deaths.

*Amputation of the arm.*—91 operations, 41 deaths, or 45 per cent., nearly one-fifth less than in amputation of the leg. Of these 91 amputations, there were for organic disease 61, and 24 deaths, or 2 in 5. The traumatic amputations were in number 30, and 17 deaths; more than one-half.

*Amputation of the fore-arm.*—28 operations, 8 deaths; 28 per cent. Of these, 17 were performed for organic disease, and 11 were traumatic; of the former, 5 died; of the latter, 3.

*Amputation at the wrist.*—The wrist was disarticulated 16 times; 12 times for chronic affections, 4 times in consequence of injuries. Of these, 4 were women and 12 men. All recovered.

*Amputation of the metatarsal bones.*—8 amputations of a single metatarsal bone were performed; one only is designated as a disarticulation. 7 of these were upon males from 8 to 41 years of age; 1 on a female of 45 years. 6 were for organic lesions; 2 were traumatic. 1 death in the second series, where the head of the bone was removed, in a man of 41 years. 4 times the first metatarsal bone was the seat of the operation; twice the fifth; once the second; in the eighth the bone is not specified.

*Amputation of the metacarpal bones.*—9 operations, limited to one of the metacarpal bones. 1 only was traumatic, and was successful; 8 were for organic lesions; among these there was 1 death in a young man of 22 years. The third metacarpal bone was amputated 6 times, the fourth once, and the first twice.

*Amputation of the great toe.*—43 disarticulations, and 7 deaths, or 1 in 6, which is enormous. 29 were from organic disease, and 14 were traumatic. Of the first series 3 died, about one-tenth; of the last, 4 died, or more than one-fourth. Of this number 5 were females, all for organic disease, and all recovered.

*Amputation of one of the toes.*—26 operations, of which 12 were traumatic, and were followed by 1 death. Of these, there were 28 males and 5 females, from the ages of 5 to 62.

*Amputation of several toes at the same time.*—In 7 operations there was 1 death.

*Amputation of one or more phalanges.*—9 operations; 4 times on the phalanx of the great toe; twice upon the phalanges of the fourth and fifth; the three others not specified. No death.

*Amputation of the Thumb.*—9 operations and 3 deaths; 3 times for organic diseases, and for a supernumerary thumb; 5 times for traumatic lesions, and of these 3 died. Three deaths in five would seem to realize for the thumb Mr. Velpeau's assertion that "amputation of the fingers is not less dangerous to life than amputation of the arm."

*Amputation of one of the fingers.*—119 operations; 109 recoveries, 10 deaths, or one-twelfth. Organic, 79; deaths, 6, or one-thirteenth; traumatic, 40; 3 deaths. Total, 1 death in 10.

*Amputation of several fingers at one time.*—13 operations; one death, in a man of forty-two years, who had had four fingers amputated.

*Amputation of the phalanges.*—This operation was performed 24 times; there was one death in a man of thirty-two years, in whom the phalanx was crushed.

Having reviewed each variety of amputation, Mr. Malgaigne proposes to examine them together, in order to deduce some general conclusions. To do this effectively, he proposes to study successively—1. The influence of the determining cause, whether pathological or traumatic. 2. The influence of sex. 3. That of age. 4. That of the season of the year; and 5. The influence of the hospitals themselves in the mortality.

In answer to the first question, *whether pathological or traumatic amputations are the most fatal*, Mr. Malgaigne's tables decide certainly in favour of the traumatic. The great pathological amputations gave 48 per cent., whilst the traumatic gave 64 per cent.; for the lesser pathological amputations the mortality was 7½ per cent. ; for the traumatic it exceeded 15 per cent. In considering the traumatic operations as primary or secondary, the data are small, there being only 26 amputations of the thigh, and 43 of the leg. Of the 26 of the thigh, 16 were primary and 10 secondary ; the first series gave 12 deaths, and the second only 6. Four of the immediate amputations were performed, however, on subjects under 20 years of age, a period at which, according to Mr. M., the mortality is greatest. All four died. The number is thus reduced to 12, and 8 deaths.

In 43 cases of amputation of the leg 33 were primary, with 22 deaths ; and 10 were secondary, with 7 deaths. Under 20 years, 5 primary amputations gave 4 deaths ; 2 secondary, 1 death. Above 20 years, there remain 28 primary amputations with 18 deaths ; whilst the secondary amputations numbered 6 deaths for 8 operations. Total,

| | |
|---|---|
| 49 primary amputations, | 34 deaths. |
| 20 secondary " | 13 " |

From our author's tables, it will be found that females resist much better than males the fatal effects of amputation. Out of 98 great pathological amputations, there were only 44 deaths; out of 40 lesser ones, 2 deaths. Of 17 great traumatic amputations there were 10 deaths ; lesser amputations, 8 deaths.

In regard to the influence which age exercises, we are at the outset struck at the great mortality which prevails between the second and fifth year, in comparison with that between the 5th and 15th year. This, M. Malgaigne thinks, is not confined to amputations, but extends to all operations. In Lithotomy he found the mortality just one-half. The data, we think, are altogether insufficient to warrant any conclusion.

In all capital operations, the season of the year has always been considered as exerting a marked influence ; and the spring and autumn were generally viewed as the most favourable periods. It appears, from the tables before us, that the greatest number of amputations are performed in the months of May, June, July, August, and September, and, moreover, that the mortality is greater in the preferred months. For the four winter months we have 81 operations and 41 deaths, and in the months of March, May, June and August, 141 operations, and 81 deaths. In repeating the calculation for each sex, from 20 to 35 years, Mr. M. finds the mortality decidedly less in winter than at any other season ; but, on the contrary, from 15 to 20, summer has incontestably the advantage. With regard to traumatic amputations the mortality is about the same for the four seasons.

With respect to the comparative mortality after amputations, in the various hospitals of Paris, our author has examined the results in nine hospitals—l'Hôtel-Dieu, la Pitié, la Charité, St. Louis, Beaujon, Neckar, St. Antoine, Cochin, and l'hôpital des Cliniques. The Hôtel-Dieu has enjoyed, for many years, the reputation of being the most unlucky of all the Parisian hospitals. For a century past it has been stated that the operation of trephining has never succeeded there, but the trephine rarely, if ever, succeeds anywhere. Within five years this operation has been performed 15 times in the hospitals of Paris, and was in every case fatal. According to Mr. Malgaigne, so far as the great amputations are concerned, the Hôtel Dieu ranks sixth in the rate of mortality, and la Charité second for the great pathological amputations, and third for the great traumatic. For the rest, the extremes only are stated in a general manner. For the pathological amputations, the most favoured hospital lost but 1 in 5, and the least fortunate lost 9 in 10; the difference being from 20 to 9 per cent. For the traumatic amputations, the most favoured had in 10 amputations 3 deaths, and the least, 5 amputations and 5 deaths; the difference being from 31 to 100 per cent. Mr. Malgaigne, in studying this part of his subject, compared first the general mortality in the same hospital, in the same series of years, with the mortality of the great amputations, but was unable to establish any ratio. He then compared the general mortality in the surgical service with that after the great amputations, but the difference in the diseases admitted into each establishment is such, that no satisfactory estimate can be made; for example, the Hôtel-Dieu is one of the hospitals in which the least deaths occur in the surgical service, whilst the hospital the most fortunate, as regards amputations, is one where the surgical mortality is the greatest.

Is the cause to be found in the amount of individual expenditure? It appears that in a period of 10 years, the most unlucky hospital was the one which expended most, and the most fortunate was the one which was the least prodigal.

Topographical position would seem to give as little aid in the solution of this problem, for the most favoured hospital, as well as the least so, are both situated on an elevation near one of the gates of the town.

An inquiry into the degree of ventilation of the different wards was not attended with more happy results. Thus frustrated in his scientific data, Mr. Malgaigne was anxious to see how far the vulgar proverb, *vieux médecin, jeune chirurgien*, was applicable. He found that the most brilliant successes, as well as the saddest reverses, attended the young surgeon, whilst the success of their seniors observed a mean. Nor does it appear that the surgeon, in changing his hospital, carries his luck, whether good or bad, with him, but bequeathes it to his successor. What, then, demands Mr. Malgaigne, is this mysterious fatality which hangs over our hospitals in general, and especial ones in particular? He imagines that, by his meditations and researches, he is able to offer a solution to the question, or, at least, to indicate some of the prominent causes.

M.r M. thinks that the lesser mortality in private practice can be accounted for better by the greater care they receive, than by the greater amount of purer air that circulates in a crowded hospital ward. Human attention, as well as reason and intelligence, has its limit, and when applied to 20 patients, each one must receive a greater share than if scattered among 40. Thus, patients in private practice are better studied than those in hospitals; and a small surgical service is, as a general rule, better visited than a large one. This view is strengthened by a reference to the mortality in the Hôpital du Midi, in which the first diminution coincided with an augmentation of the number of physicians; and the second with the distribution of the sexes in different hospitals; and receives additional confirmation from the other hospitals of Paris, particularly the Hôtel-Dieu. When Dupuytren alone had charge of the whole surgical service, the mortality was:

| | |
|---|---|
| In 1818, | 1 in 10. |
| In 1820, | 1 in 13. |
| In 1821, | 1 in 15. |
| In 1822, | 1 in 11. |
| In 1828, | 1 in 16. |

Compare these results with those since the division of the service:

| | |
|---|---|
| In 1837, | 1 in 18. |
| In 1838, | 1 in 19. |
| In 1839, | 1 in 19. |
| In 1840, | 1 in 17. |

Although this might be thought to furnish sufficient indirect proof against too large wards, yet more precise data can be further alleged. At the period of the invasion, the hospitals being insufficient to accommodate all the wounded soldiers, the Prefect of the Seine placed at the disposition of the Conseil des Hôpitaux, the *abattoirs* of Roule, Montmartre, and Ménilmontant. These public slaughter houses are composed of distinct pavillions, separated by large streets, and are of one story. A comparison of the mortality in these and in the hospitals, results considerably in favour of the abattoirs.

The diet of the patients also exercises, Mr. Malgaigne thinks, a decided influence. During the period above mentioned, the French and German soldiers had the same regimen, whilst the Russians were rarely placed on a soup diet, and hardly ever on *diéte* absolue. In consequence, those who were slightly wounded received a full portion, and the others a half portion.*

* In the 4th vol. of Guy's Hospital Reports, Sir Bransby Cooper, in an article on amputation, states that he has been credibly informed that not more than three or four patients out of one hundred, who undergo this operation in the Parisian Hospitals, are saved; and he adds his conviction that this extraordinary mortality is in a great measure dependent upon the meagre diet of the patients, and to the use of

The proportion of deaths is thus stated :

| | |
|---|---|
| French soldiers, | 1 in 7. |
| Prussian " | 1 in 9. |
| Austrian " | 1 in 11. |
| Russian " | 1 in 26. |

Another circumstance would seem also to have diminished the mortality among the Russians—the use of the vapour baths. To judge from the following statement, their influence was very great. In two hospitals without baths, the mortality was 1 in 10, and 1 in 11 ; in the two with baths, 1 in 18, and 1 in 77.

Mr. Malgaigne concludes thus : " The impression which this examination has made upon me has been a sorrowful one. The successes of modern surgery, estimated by this terrible scale of mortality, appear far less brilliant; and yet I have rather softened the results. Thus I have counted as cured, all amputations which did not die; and it was necessary that these supposed cures should all be counted as triumphs of our art. More than one amputation was but imperfectly cicatrised ; more than one patient left the hospital prematurely, to return afterwards and undergo new operations, and often to die. I could not omit this last character of so sad a picture ; it was necessary that I should unveil this deep and unsuspected wound of our surgery ; now the masters are warned, averted, and put on their guard : *Cureant consules ne quid respublica detrimenti capiat.*"

Our intention was to have compared the above results with those obtained in this country and England, but our limits oblige us to postpone this to a succeeding number. M. Clymer.

the *vin ordinaire*, as the only stimulus employed ; a beverage, he adds, totally insufficient for the purpose. How correct the first part of the statement is, the present article enables our readers to judge. In regard to the second question we may state, after a constant attendance for a long period in the hospitals of Paris, that the diet allowed is of an excellent and nutritious quality, and sufficient in quantity ; and, moreover, that in all cases where the surgeon deems the exhibition of stimulants demanded, they are freely given.

